# Tunable Open Circuit Voltage by Engineering Inorganic Cesium Lead Bromide/Iodide Perovskite Solar Cells

**DOI:** 10.1038/s41598-018-20228-0

**Published:** 2018-02-06

**Authors:** Chi Huey Ng, Teresa S. Ripolles, Kengo Hamada, Siow Hwa Teo, Hong Ngee Lim, Juan Bisquert, Shuzi Hayase

**Affiliations:** 1 Department of Chemistry, Faculty of Science, Universiti Putra Malaysia, 43400 UPM Serdang, Selangor Malaysia; 2 Graduate School of Life Science and Systems Engineering, Kyushu Institute of Technology, 2-4 Hibikino, Wakamatsu-ku, Kitakyushu 808-0196 Japan; 3 Functional Device Laboratory, Institute of Advanced Technology, Universiti Putra Malaysia, 43400 UPM Serdang, Selangor Malaysia; 4 Institute of Advanced Materials (INAM), Universitat Jaume I, 12006 Castelló, Spain; 5 Department of Chemistry, Faculty of Science, King Abdulaziz University, Jeddah, Saudi Arabia

## Abstract

Perovskite solar cells based on series of inorganic cesium lead bromide and iodide mixture, CsPbBr_3-*x*_I_*x*_, where *x* varies between 0, 0.1, 0.2, and 0.3 molar ratio were synthesized by two step-sequential deposition at ambient condition to design the variations of wide band gap light absorbers. A device with high overall photoconversion efficiency of 3.98 % was obtained when small amount of iodide (CsPbBr_2.9_I_0.1_) was used as the perovskite and *spiro*-OMeTAD as the hole transport material (HTM). We investigated the origin of variation in open circuit voltage, *V*_oc_ which was shown to be mainly dependent on two factors, which are the band gap of the perovskite and the work function of the HTM. An increment in *V*_oc_ was observed for the device with larger perovskite band gap, while keeping the electron and hole extraction contacts the same. Besides, the usage of bilayer P3HT/MoO_3_ with deeper HOMO level as HTM instead of *spiro*-OMeTAD, thus increased the *V*_oc_ from 1.16 V to 1.3 V for CsPbBr_3_ solar cell, although the photocurrent is lowered due to charge extraction issues. The stability studies confirmed that the addition of small amount of iodide into the CsPbBr_3_ is necessarily to stabilize the cell performance over time.

## Introduction

Perovskite solar cells have attracted great attention as the prime energy conversion devices, owing to its reasonably low fabrication cost without compromising its photovoltaic performances. Relentless efforts have been done to optimize the parameters of perovskite solar cell for high power conversion efficiency (PCE) purpose, typically the hybrid organic-inorganic methylammonium lead halide (CH_3_NH_3_PbX_3_ or MAPbX_3_, where X is a halide Cl, Br, I, or mixture of halides) which has accomplished PCE over 20%^1–3^. Particularly, the mixture of halides in perovskite solar cells were envisioned to be able to adjust the absorption spectra which may be an advantageous property for full spectrum energy harvesting and photoelectrochemical applications. By tuning the iodide/bromide ratio of the mixed halides perovskite solar cell such as the increment of iodide concentration led to red-shifting of the absorption range and thereby, enhances the photovoltaic performance. Under this scenario, Seok and co-workers reported a PCE of 12.3 % for the MAPb(I_1-*x*_Br_*x*_)_3_ perovskite solar cell and manifested the feasibility in obtaining excellent photovoltaic performances by manipulating the ratio between halides of I^−^ and Br^−^^4^. Similar performances were also obtained from Huang and co-workers for an inverted MAPb(I_1-*x*_Br_*x*_)_3_ architecture, using a bilayer of indene-C_60_ tri-adducts (ITCA) and C_60_ as electron transport materials^5^. Even, hybrid iodide/bromide perovskite hole conductor free solar cells showed good stability and efficiencies of 8.54 %^6^. In addition, a MAPb(I_1-*x*_Br_*x*_)_3_ solar cell prepared through vacuum deposition technique by Bolink and co-workers achieved an efficiency of 10.2 %^7^. However, aforementioned perovskite possesses restriction on stability and therefore, researchers replace the conventional organic cation (MA^+^) to inorganic cation such as cesium (Cs^+^). Thermogravimetric analyses confirm that the inorganic perovskite, cesium lead bromide (CsPbBr_3_) has higher thermal stability than the hybrid organic-inorganic perovskite, MAPbBr_3_^8^. Additionally, CsPbBr_3_ exhibits good charge transport properties with an electron mobility of ~1000 cm^2^/Vs and electron lifetime of 2.5 μs^9,10^. Whereas, CsPbI_3_ perovskite solar cells show enhancement in optical and electrical properties^11^. So far, the photoconversion efficiencies of both perovskite solar cells are poor due to low charge carrier collection of CsPbBr_3_^8^, and structural changes of CsPbI_3_ perovskite at room temperature. Despite CsPbI_3_ perovskite solar cell fabricated under vacuum-processed has achieved high efficiency of 10.5 %, subsequently the improved efficiency of mixed halides or mixed halide-mixed Pb/Sn perovskite solar cells up to 11 % has been reported, however, the need of high technology equipment such as vacuum chamber and glove box during the fabrication process have restrained its outdoor applications competency owing to unsolved stability issue and high production cost^12–15^. Therefore, a mixture of halides cesium perovskite absorber material, CsPbBr_3-*x*_I_*x*_ is a suitable harvester candidate to improve the optical and electrical properties, subsequently enhances the cell performance and stability performance.

Generally, high open circuit voltage *V*_oc_ is one of the photovoltaic parameters to be achieved for high overall photoconversion efficiencies. The factors that control the *V*_oc_ are the adequate match of the Lowest Unoccupied Molecular Orbital (LUMO) level of the electron transport material (ETM) and the conduction band of the perovskite layer, as well as the Highest Occupied Molecular Orbital (HOMO) level of the hole transport material (HTM) and the valence band of the perovskite layer^16^. However, other studies predicted that the *V*_oc_ is closely related to the charge recombination rate and the work functions of the HTMs^17–19^. An example that confirms this theory where the *V*_oc_ of TiO_2_/MAPbI_3_ layer covered with copper iodide (CuI) as the *p*-type semiconductor was reduced, as opposed to other devices without this layer. This behavior was attributed to higher recombination processes^20^. Other reports also confirm that the *V*_oc_ is solely determined by the perovskite absorber, regardless of the TiO_2_ surface modifications such as TiO_2_-coating with a thin film of MgO in order to change the conduction band^21^. Furthermore, an inverted perovskite solar cell using conducting polymers as the hole-selective contact materials exhibited direct correlation between higher work functions of HTM and lower charge recombination rate, consequently the *V*_oc_ was increased^17,19^. Interesting results were recently obtained for MAPbBr_3_-based solar cells with mesoporous-TiO_2_ scaffold layer. Two HTMs, a novel molecule based on 1,3,4- oxadiazole ring (H1) and the standard *spiro*-OMeTAD with different HOMO levels of −5.43 and −5.22 eV, respectively were compared. The device prepared with H1 achieved a *V*_oc_ of 1.43 V and was further increased to 1.49 V with the usage of *spiro*-OMeTAD for solar cell devices. The authors confirm that there was not a clear correlation between HOMO level of the MAPbBr_3_ (−5.9 eV) and work function of the HTMs^22^. Therefore, in-depth study to further improve the *V*_oc_ and efficiency performance is still continuing. However, it is notable that the *V*_oc_ is originated from two factors, which are the band gap of the perovskite material and the perfect matching of electron and hole transporting interface.

In this manuscript, we combine two important properties in achieving high *V*_oc_ in standard solar cells, which are the tunable band gap in inorganic perovskite absorbers and the capability to extract hole charge carrier properly. A number of CsPbBr_3-*x*_I_*x*_ perovskite absorber films were synthesized through two-step deposition technique by varying small amount of *x* ratio from 0 to 0.3. These films were characterized by X-ray diffraction (XRD), UV-Vis spectroscopy, photoluminescence, and ultraviolet photoelectron spectroscopy (UPS). Upon the addition of iodide into the perovskite CsPbBr_3_ matrix, the band gap descended and consequently, the *V*_oc_ decreased. In addition, the HTMs pose direct effect on the *V*_oc_ behavior. The pure CsPbBr_3_ solar cells reported high *V*_oc_ close to 1.16 V with *spiro*-OMeTAD, but increased to 1.30 V with a bilayer of P3HT/MoO_3_. Finally, the stability issues were also analyzed in this work, concluding that small amount of iodide indeed stabilize all photovoltaic parameters.

## Results and Discussion

### Absorption, emission, and band gap

**Figure 1:**
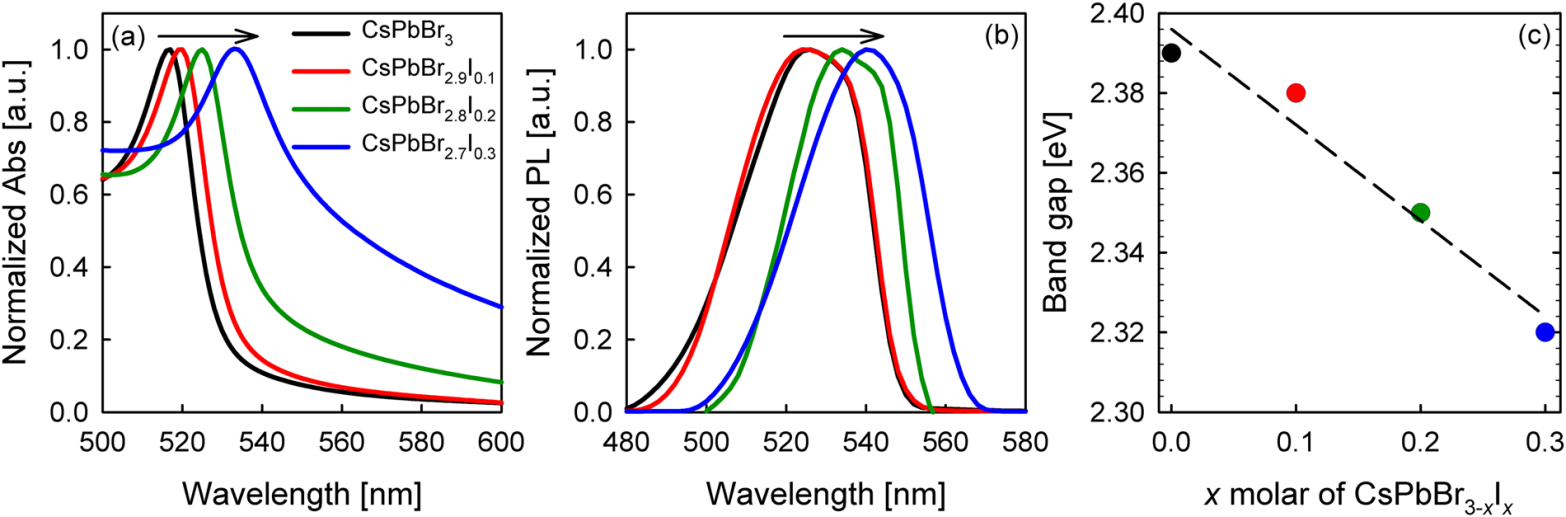
Normalized (**a**) absorption (Abs) and (**b**) photoluminescence (PL) of CsPbBr_3-*x*_I_*x*_, where *x* is 0, 0.1, 0.2, and 0.3 in molar ratio. The intersection between Abs and PL spectra represents the (**c**) band gap energy.

**Figure 2:**
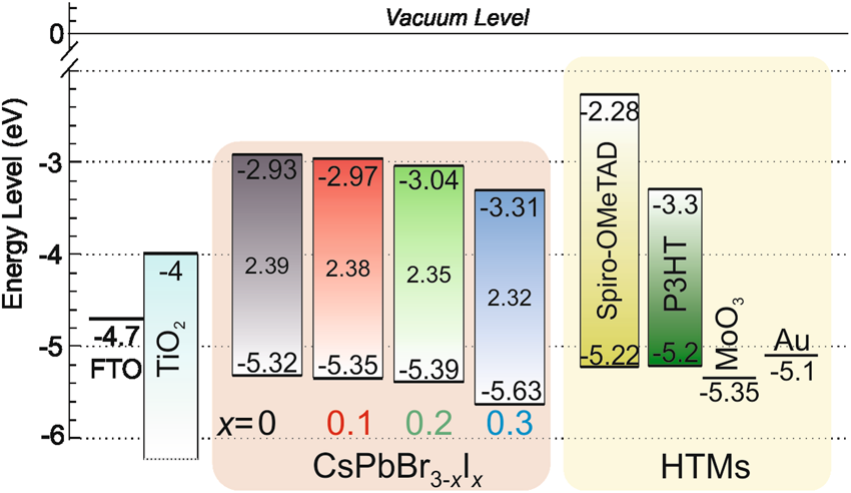
Schematic energy level diagram of a complete TiO_2_-mesoporous CsPbBr_3-*x*_I_*x*_ (*x* is 0, 0.1, 0.2 or 0.3) perovskite solar cell, employing either *spiro*-OMeTAD^34^ or P3HT/MoO_3_^32,35^ as the hole transporting material. The band gap of each perovskite, where *x* is 0, 0.1, 0.2, and 0.3 was also indicated.

CsPbBr_3-*x*_I_*x*_ perovskites (*x* varies from 0 to 0.3) were synthesized through two-step sequential deposition process by spin coating different molar ratio of PbI_2_:PbBr_2_ solution, subsequently dipped in CsBr solution for perovskite formation in ambient condition. Figure 1 shows the UV-visible absorption and emission spectra of the CsPbBr_3-*x*_I_*x*_ perovskite films with different bromide/iodide molar ratio. Both the absorption and emission spectra display red-shifted trend towards longer wavelength as the iodide concentration of the CsPbBr_3-*x*_I_*x*_ perovskite films increases, as illustrated in Figure 1a and 1b. The lower absorption coverage of a purely bromide film could be ascribed to the bromide ion, which narrows the vibration mode and band near-IR region^23^. The band gap (*E*_*g*_) of each perovskite material was calculated via equation (1),

1 $${E}_{{\rm{g}}}=\frac{1240}{\lambda }$$

where *E*_*g*_ refers to the band gap and *λ* is the intersected wavelength of the UV-Vis and photoluminescence. To be specific, the wavelength obtained from the intersection point between UV-Vis and photoluminescence was inserted into equation (1) in determining the band gap of each perovskite material, as summarized in Figure 1c. When the amount of PbI_2_ in CsPbBr_3_ matrix increases, gradual reduction in *E*_g_ was observed from 2.39 (CsPbBr_3_), 2.38 (CsPbBr_2.9_I_0.1_), 2.35 (CsPbBr_2.8_I_0.2_), to 2.32 eV (CsPbBr_2.7_I_0.3_), which is in great agreement with the previous reports in the literature^4,24^. In order to complete the energy level diagram, the valence band of each CsPbBr_3-*x*_I_*x*_ perovskite material, *x* = 0, 0.1, 0.2, and 0.3, respectively, was measured through ultraviolet photoelectron spectroscopy (UPS), as shown in Figure S1. The HOMO levels have recorded the values of −5.52, −5.35, −5.60, and −5.61 eV, corresponding to CsPbBr_3-*x*_I_*x*_, where *x* = 0, 0.1, 0.2, and 0.3, respectively, as manifested in Figure 2.

### Crystallinity and morphological structure

The crystallinity of the perovskite films was investigated via XRD to study the crystal phase transition of the perovskite materials. The temperature dependent CsPbX_3_ perovskite material could be existed in three polymorphic structures, either in tetragonal, orthorhombic, or in cubic structure. Interestingly, at high annealing temperature of 350 °C, the transformation of CsPbX_3_ material into cubic phase was observed from the XRD profile (Figure S2). The experimental peaks are closely matches the theoretical CsPbBr_3_ peaks (JCPDS file #00–054–0752) and are in agreement with other reported works in the literature^8,25,26^. The highly crystalline peaks at 15.2°, 21.6°, and 30.6° are indexed to (100), (110), and (200), respectively. In addition, some additional low intensity peaks detected from 22° to 30° are corresponded to the non-reacted PbBr_2_. The intensity of the PbBr_2_ peaks was diminished and even disappeared owing to the PbBr_2_ has been completely substituted by PbI_2_ when the concentration of iodide increases. The presence of low intensity peak of CsBr was also detected at 30° in all perovskite films due to synthesis of perovskite film was through two-step sequential deposition. The XRD profile explicates an unnoticeable blue-shifted trend to lower angles region as the concentration of iodide increases. This is owing to the substitution of larger iodide ions over the bromide lattice, which expanded the d-spacing of the halide matrix and thus inversely brought to lower degree detection^14^. Figure S3 shows the FE-SEM images of CsPbBr_3-*x*_I_*x*_ perovskite films. Pinholes (yellow arrow) are observed on the surface of CsPbBr_3_ perovskite film where the formation of voids could be due to evaporation of organic solvent during annealing process. Whereas, upon the inclusion of iodide anion, the compactness of the perovskite is gradually improved with the presentation of pinhole-free surface, as shown in Figure S3(b–d). Figure S3d shows the alteration of perovskite structure of CsPbBr_2.__7_I_0.3_ (observation of non-homogenized morphological surface) when 0.3 molar ratio of iodide was incorporated within bromide matrix, which could be due to the overloading of iodide anion.

### Photovoltaic performances

**Figure 3:**
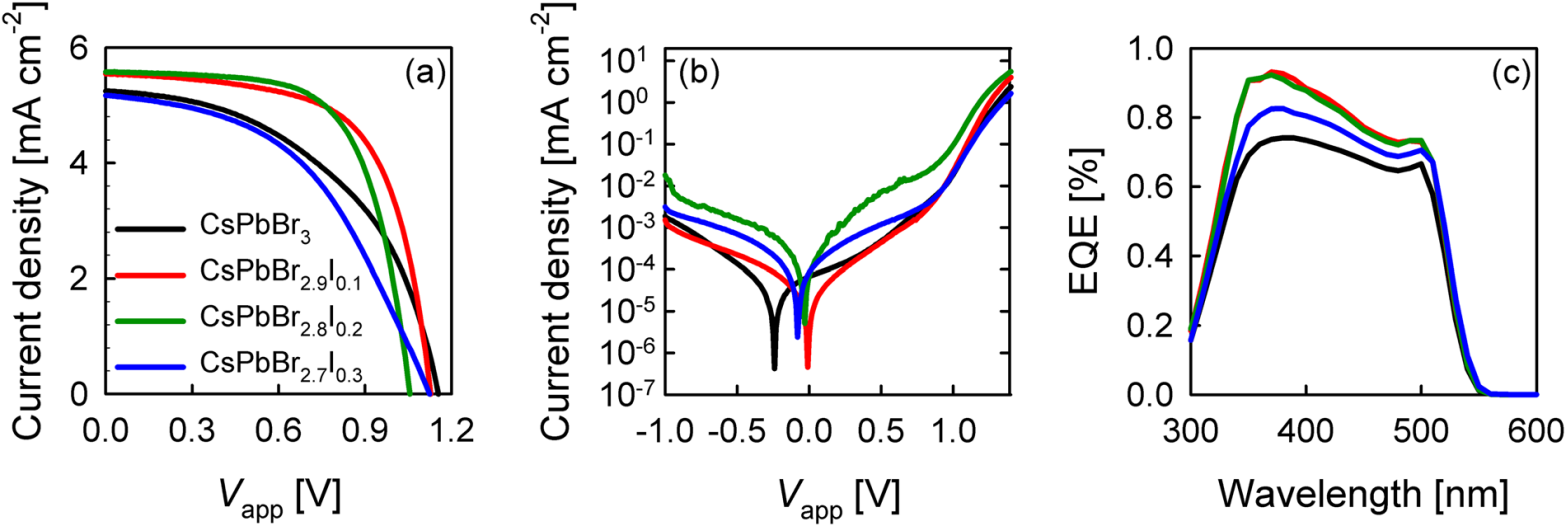
(**a**) J-V measurements under simulated AM1.5G sun light of 100 mW cm^−2^ irradiance, and (**b**) under dark. (**c**) Represents the EQE for glass/FTO/c-TiO_2_/mp-TiO_2_/CsPbBr_3-*x*_I_*x*_/*spiro*-OMeTAD/Au solar cells, where *x* varies in 0, 0.1, 0.2, and 0.3 molar ratio.

The photovoltaic performances of different bromide/iodide ratios solar cell, employing CsPbBr_3-*x*_I_*x*_ (*x* varies from 0, 0.1, 0.2, and 0.3 molar ratio) as the perovskite layer and *spiro*-OMeTAD as the HTM were measured under 1 sun (AM1.5G 100 mWcm^−2^) illumination and dark conditions, as depicted in Figure 3 and the corresponding photovoltaic results were tabulated in Table 1.

**Table 1:** Photovoltaic parameters of the CsPbBr_3-*x*_I_*x*_ solar cells (*x* varies between 0, 0.1, 0.2, and 0.3 in molar ratio) with *spiro*-OMeTAD as the HTM.

Perovskite	*V*_oc_ (V)	*J*_sc_ (mA/cm^2^)	*FF*	PCE (%)	*J*_sc_[Cal.] (mA/cm^2^)	*R*_sh_, kΩ/cm^2^	*R*_s_, Ω/cm^2^	HI
CsPbBr_3_	1.16	5.25	0.49	2.97	5.85	220.3	2711	0.11
CsPbBr_2.9_I_0.1_	1.13	5.54	0.64	3.98	6.83	294.5	1674	0.04
CsPbBr_2.8_I_0.2_	1.06	5.58	0.65	3.83	6.78	673.0	1724	0.0002
CsPbBr_2.7_I_0.3_	1.12	5.17	0.47	2.73	6.44	158.5	5194	0.08

The photocurrent calculated from the integration of the EQE curves was also added. Hysteresis index (HI) was calculated by equation (2).

Taking into account of all photovoltaic parameters, the addition of minute amount of iodide has dramatically improved the photoconversion efficiency of CsPbBr_3_ from 2.97 % to 3.98 % for CsPbBr_2.9_I_0.1_ solar cell with high open circuit voltage (*V*_oc_) of 1.13 V and fill factor (*FF*) of 0.64. Similar photovoltaic results were also observed for CsPbBr_2.8_I_0.2_ device. Nevertheless, further increasing the iodide amount in CsPbBr_3-*x*_I_*x*_ did not improve the cell performance. Contrary, the photoconversion efficiency plunged to 2.73 % when 0.3 molar ratio of iodide was incorporated to the bromide matrix. The performance differences were dependent on the short circuit current (*J*_sc_) and *FF*; while keeping the *V*_oc_ almost constant (Figure 3a). The substantial reduction of *FF* for CsPbBr_2.7_I_0.3_ solar cell could be attributed to the occurrence of minor interfacial charge recombination, which resulted from relatively higher series and lower shunt resistances, as manifested in Table 1. The J-V curves under dark at −1 V reflected small differences between devices (Figure 3b) implies suppressed charge recombination^27^. The *J*_sc_ and *FF* parameters were improved when the iodide concentration was either in 0.1 or 0.2 molar ratio (CsPbBr_2.9_I_0.1_ or CsPbBr_2.8_I_0.2_) relative to CsPbBr_3_ and CsPbBr_2.7_I_0.3_ perovskite solar cells. These results imply that higher amount of iodide disturbs the charge extraction of the cell and thus only an optimized amount of PbI_2_ is favorable. Figure 3c shows the external quantum efficiency (EQE) spectra of CsPbBr_3-*x*_I_*x*_ devices and the results are consistent with the *J*_sc_ performance. The EQE intensity increases from 350 nm to 500 nm upon the addition of iodide into the pure CsPbBr_3_. The current calculated from the integration of the EQE curves demonstrated the occurrence of some recombination mechanisms that affect these devices, consequently reduced the *J*_sc_ performance. Figure S4 shows the enlarged EQE spectrum of CsPbBr_3-*x*_I_*x*_ based perovskite devices. It can be seen that there are noticeable shifts of EQE onset to longer wavelengths in conjunction with the iodide increment, which is in great agreement with the absorption spectra in Figure 1.

Hysteresis effects are first to be investigated for cesium-based perovskite solar cell with mixed halide architecture, as manifested in Figure S5. The hysteresis index (HI) was obtained via equation (2)^11,28^.

2 $$\frac{{J}_{{\rm{RS}}}(0.5{V}_{{\rm{oc}}})-{J}_{{\rm{FS}}}(0.5{V}_{{\rm{oc}}})}{{J}_{{\rm{RS}}}(0.5{V}_{{\rm{oc}}})}$$

where *J*_RS_(0.5*V*_oc_) and *J*_FS_(0.5*V*_oc_) represent the photocurrent density at 50% of *V*_oc_ for the reverse (RS) and forward (FS) scan directions, respectively. As a reference HI is 0 when no hysteresis was observed, while HI of 1 represents the case where the hysteresis is as high as the magnitude of the photocurrent. It shows that the hysteretic performance of the CsPbBr_3-*x*_I_*x*_, where *x* = 0.1–0.3 perovskite devices is alleviated with the inclusion of iodide, as compared to the purely CsPbBr_3_ device with a HI value of 0.11. Considering the hysteretic performance of the champion cell (CsPbBr_2.9_I_0.1_) against CsPbBr_3_, hysteresis index was doubly reduced to 0.04 when 0.1 molar ratio of iodide was incorporated. It hence implies that mitigation of hysteretic effect can be done not only through cation engineered, but also through halides architecture, which should be the next research focus.

**Figure 4:**
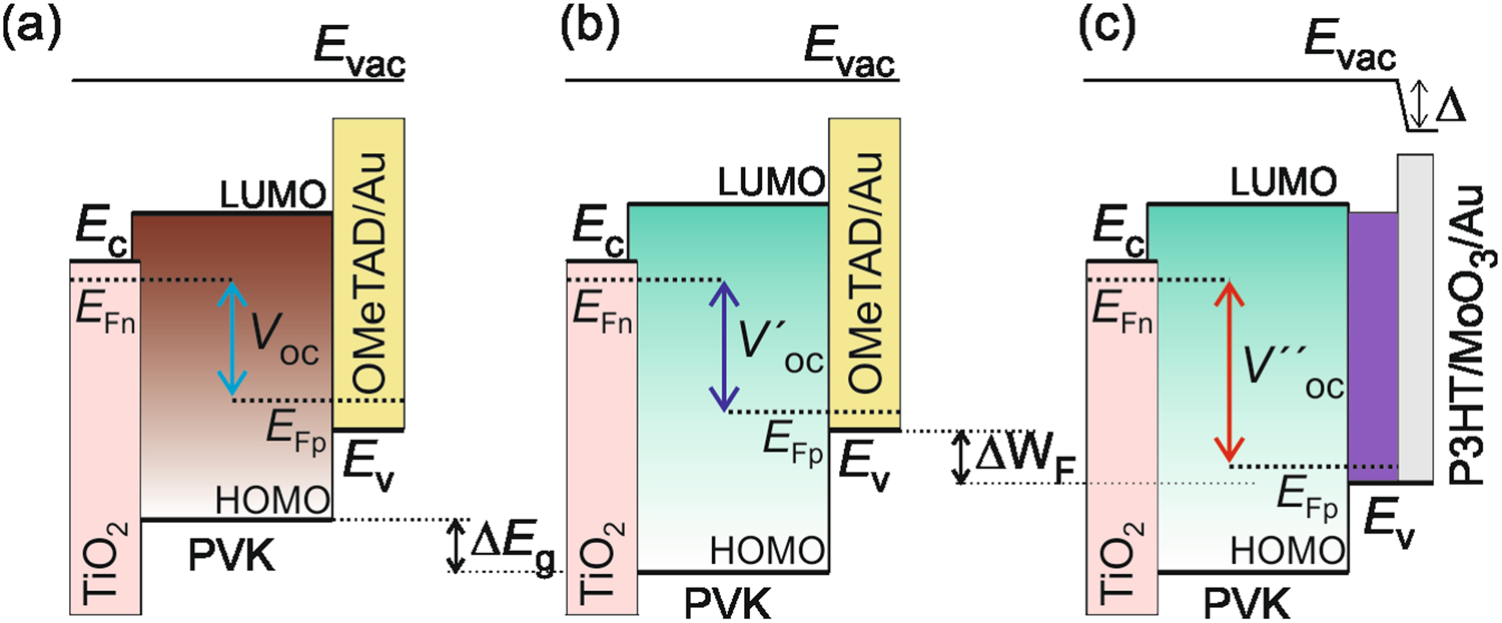
The *V*_oc_ behavior of the perovskite solar cells is represented in the energy level diagrams by employing (**a**) narrow or (**b**) wide band gaps (*E*_g_) perovskite absorber, and (**c**) different HTMs, such as *spiro*-OMeTAD/Au in (**a**) and (**b**) or deeper work function (W_F_) P3HT/MoO_3_/Au in (**c**). The conduction band (*E*_c_), valence band (*E*_v_), Fermi level of electrons (*E*_Fn_) and holes (*E*_Fp_) were indicated. The MoO_3_ layer creates a dipole (Δ) at the interface, reducing the vacuum level energy (*E*_vac_). The Au performs as the electron blocking layer.

There are two factors that are able to tune the *V*_oc_ performance of a perovskite solar cell, which are the band gap of the halide mixture perovskite material and the employment of different work function HTM, as manifested in Figure 4. Analyzing solar cells of different perovskite band gap, the *V*_oc_ should be improved for the solar cells with larger perovskite band gap rather than narrower band gap. Figure 4a and 4b illustrated the *V*_oc_ behavior of a perovskite solar cell towards a narrower and a wider perovskite band gap, respectively. It shows that small *V*_oc_ increment was observed when a device with larger perovskite band gap was used; taking the hole and electron transport materials the same. Due to the addition of minute amount of iodide within the CsPbBr_3-*x*_I_*x*_ matrix, and a consequence of small shift on band gap (Δ*E*_g_), the *V*_oc_ changed slightly under these circumstances.

Despite the photovoltaic devices employing *spiro*-OMeTAD as the HTM performed high *V*_oc_ close to 1.1 V, nevertheless, it was expected to achieve even higher *V*_oc_ values with the usage of wide band gap perovskite materials. To gain further insight on *V*_oc_ tuning and subsequently, the performance of the photovoltaic device, the choice of hole extractor is an another factor to be considered besides the perovskite absorbers. When a bilayer polymer/oxide (P3HT/MoO_3_) was used as the HTM, the *V*_oc_ was drastically improved from 1.16 V to 1.3 V, as manifested in Figure S6 and Table S1 owing to deeper HOMO energy level of P3HT/MoO_3_ than *spiro*-OMeTAD. The offsets of HOMO level between P3HT-perovskite in the range of 0.1–0.4 eV merited the hole extraction process. Moreover, the inclusion of MoO_3_ interlayer between P3HT and Au performs as exciton blocking layer, herein retards reverse charge flow^29^. Figure 4c shows that the P3HT-based solar cells with the same perovskite materials shifted the hole Fermi level (*E*_Fp_) to lower energy level, hence enhanced the *V*_oc_ considerably. In all cases, the *V*_oc_ enhancement was observed for the P3HT/MoO_3_ devices, as compared to the *spiro*-OMeTAD devices, except for CsPbBr_2.7_I_0.3_ solar cell. This situation is similar to the large *V*_oc_ increment obtained in dye-sensitized solar cells when using redox couple with more positive redox potential^30^. Figure S6c illustrates the EQE spectra of CsPbBr_3-*x*_I_*x*_ devices using P3HT/MoO_3_ as the HTM were correlating well with the *J*_sc_ performances. All in all, it thus confirmed that the origin of the *V*_oc_ is depending on both the band gap of the perovskites and the work function of the HTMs. Photovoltaic performances of champion CsPbBr_3-__*x*_I_*x*_ solar cells and its average photovoltaic performances of five solar devices, coupled with the error bars are depicted in Figure S7 and Table S2.

**Figure 5:**
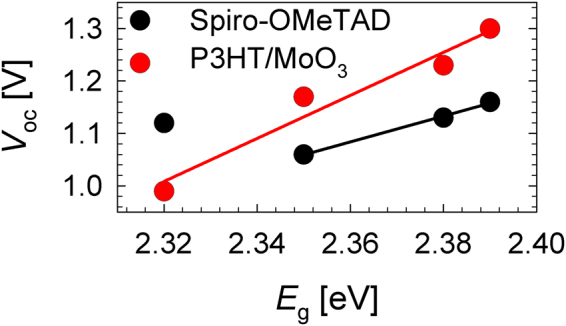
Band gap dependence of open circuit voltage in devices with different CsPbBr_3-*x*_I_*x*_ perovskite compositions and HTM such as *spiro*-OMeTAD or P3HT/MoO_3_.

Figure 5 shows the linear dependency between *V*_oc_ and band gap (*E*_g_) of CsPbBr_3-*x*_I_*x*_ perovskite absorbers using different HTMs, *spiro*-OMeTAD and P3HT/MoO_3_. This pattern is more pronouncing for the case of P3HT because P3HT has a deeper HOMO level than *spiro*-OMeTAD. Nevertheless, the CsPbBr_2.7_I_0.3_-based solar cell with *E*_g_ of 2.32 eV did not follow the mentioned tendency, which may be due to the HOMO level collected from the UPS is much deeper in energy level (−5.61 eV) than the other CsPbBr_3-*x*_I_*x*_ perovskites and thus was excluded from the linear fitting of Figure 5.

**Figure 6:**
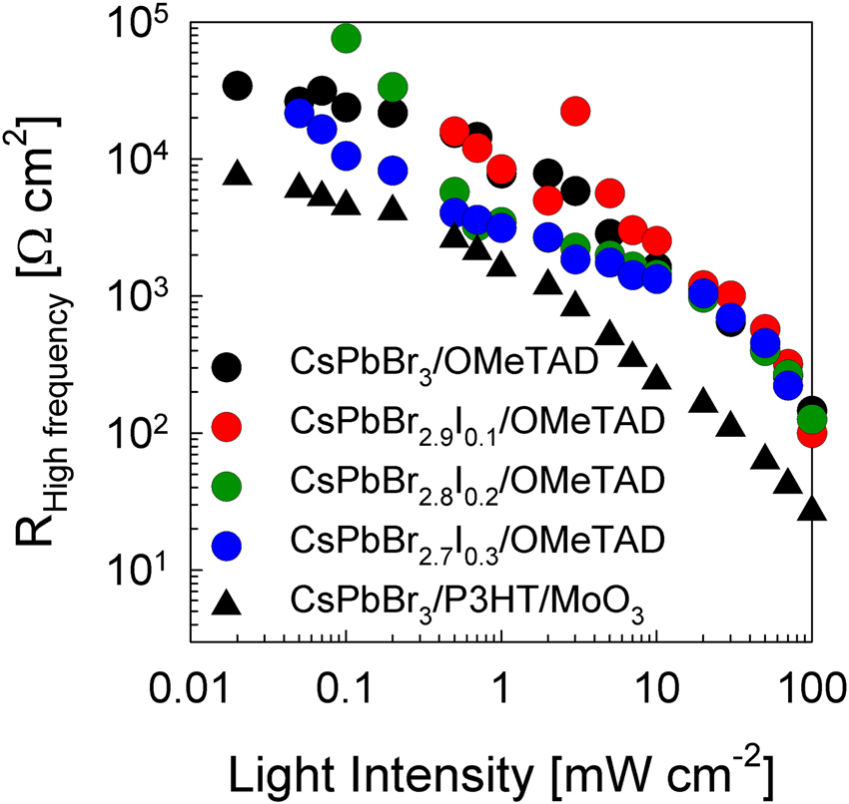
Resistance fitted at high frequency range in impedance spectroscopy measured at numerous light intensities from low light intensity to 1 sun illumination. Different CsPbBr_3-*x*_I_*x*_ perovskite compositions (*x* varies between 0, 0.1, 0.2, and 0.3 in molar ratio) and hole transport materials as *spiro*-OMeTAD/Au (circle dots) and P3HT/MoO_3_/Au (triangle dots) were analyzed.

There is another point of view to explain the photovoltaic response of these materials. A solar cell is composed of sequence of materials, carefully chosen with different work functions in order to extract the photoinduced charge carrier efficiently. Thus, larger differences in the work function of P3HT/MoO_3_, as compared to the *spiro*-OMeTAD using the same perovskite layer for both cases were studied. The first case (*spiro*-OMeTAD) enabled efficient charge extraction and contributed to higher *V*_oc_. However, the P3HT/MoO_3_ devices showed reduction in *J*_sc_ in all solar cells (Figure S6a and Table S1) could be due to shorter electron lifetime of P3HT based solar cells than *spiro*-OMeTAD based solar cells. To further understand this phenomenon, additional charge loss processes at the interface of perovskite/HTM were found in the impedance spectroscopy results. Figure 6 displays the fitted resistance at different illumination intensities from 0.02 to 100 mWcm^−2^ radiation. This measurement was carried out at high frequency range in order to avoid polarization and memory effects. The equivalent circuit model to fit the impedance plot, one arc, was a simple resistance in parallel with a capacitance. Close to 1 sun illumination intensity, all CsPbBr_3-*x*_I_*x*_/*spiro*-OMeTAD solar cells showed the same resistance pattern, independent of the composition of the perovskite layer. Small amount of iodide unaltered the above resistance. However, when the HTM was changed to P3HT/MoO_3_ for CsPbBr_3_-based solar cell, the resistance dropped considerably at the same charge carrier or light intensity. This effect was correlated to the reduction of the *J*_sc_ observed in the J-V curves. This behavior means that the resistance at the interface between perovskite/HTM detected variations when the contact changed, namely *spiro*-OMeTAD and P3HT/MoO_3_, but was constant when the perovskite composition (CsPbBr_3-*x*_I_*x*_) changed. As a matter of fact, it was observed in Figure 2 that the effective LUMO of P3HT/MoO_3_ was rather close to perovskite absorber *E*_v_ energy, indicating that the driving force for hole carrier extraction was small. This feature lead to charge accumulation at the interface, which increases the recombination and thereby, the loss of photocurrent occurred (Figure S6a).

**Figure 7:**
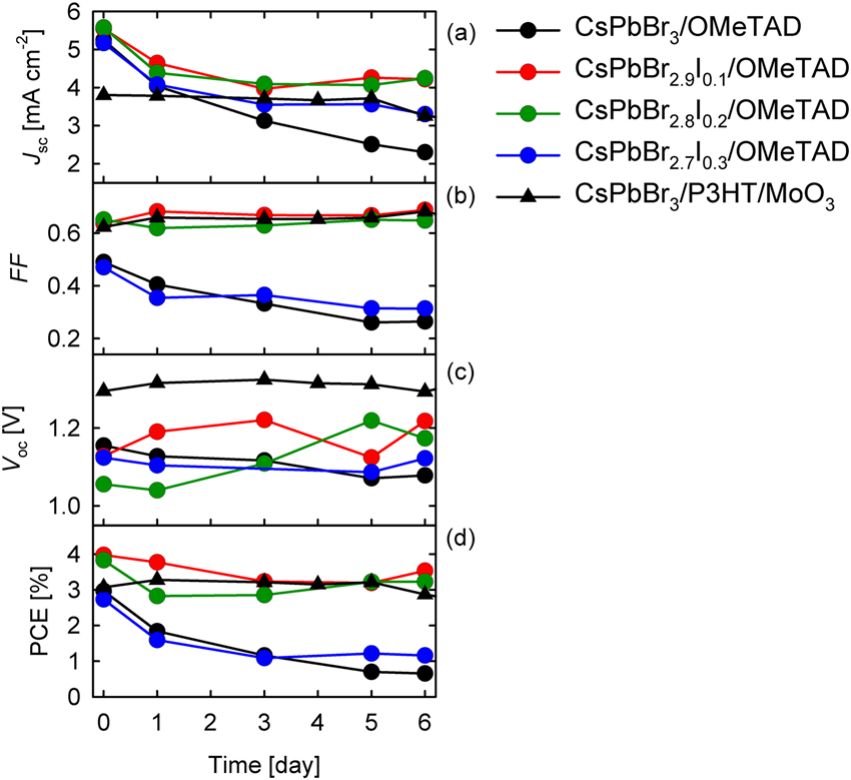
Aging analysis of CsPbBr_3-*x*_I_*x*_ devices (*x* varies between 0, 0.1, 0.2, and 0.3 molar ratio). Figures show the cell parameters (**a**) *J*_sc_, (**b**) *FF*, (**c**) *V*_oc_, and (**d**) efficiency as a function of time.

Another important factor to be studied is the stability performance of the devices when a small amount of PbI_2_ was added to synthesize CsPbBr_3-*x*_I_*x*_ perovskite solar cells. The measurements were carried out in ambient air with a thin film of polymethylmethacrylate, PMMA, deposited on the Au surface for encapsulation purpose. Thus, oxygen and water factors were to be omitted. The performance was measured for six days and between measurements, the devices were kept in the glove box under nitrogen ambient and dark conditions. Aging analysis of CsPbBr_3-*x*_I_*x*_ solar cells are summarized in Figure 7.

The CsPbBr_3_- and CsPbBr_2.7_I_0.3_-based devices showed steady decay in *FF* and more pronounced in *J*_sc_ with constant *V*_oc_. The decrease of *J*_sc_ for CsPbBr_2.9_I_0.1_ and CsPbBr_2.8_I_0.2_ solar cells was less drastic, whilst the *FF* and *V*_oc_ remain constant. There are several reasons to explain the reduction of *J*_sc_, as observed in all devices. The *spiro*-OMeTAD solution used as a HTM was composed of 4-*tert*-butylpyiridine as an additive, which may react with the perovskite layer^31^. Another additive used in the previous solution was *bis* (trifluoromethane) sulfonimide lithium salt (Li-TFSI) which increases the hole mobility and conductivity, but the cell stability can be affected by the oxidation process^32^. Or even, some studies confirm that the mesoporous-TiO_2_ created additional deep traps due to oxygen desorption at the surface caused by the UV-illumination^33^. It is important to highlight that small addition of iodide into the CsPbBr_3_ not merely improved the photoconversion efficiencies from 0.66 % for CsPbBr_3_ to 3.53 % for CsPbBr_2.9_I_0.1_ after 6 days but also its stabilities. Contrary, high amount of PbI_2_ such as CsPbBr_2.7_I_0.3_ perovskite damaged the absorber composition over time, resulted an efficiency of 1.16 % after 6 days. Other important factor to consider is the selective HTM in perovskite solar cells. The P3HT/MoO_3_/Au layer acted as a stabilizer in the solar cells, keeping all photovoltaic parameters constant over time. The large *V*_oc_ (>1.3 V) achieved for the CsPbBr_3_/P3HT/MoO_3_-based solar cell highlights that this material is promising for its numerous applications.

## Conclusions

In summary, we reported a complete film and device characterization for CsPbBr_3-*x*_I_*x*_ perovskite where *x* varies between 0, 0.1, 0.2, and 0.3 molar ratio, and the absorbers fall in large band gap’s category, specifically more than 2.3 eV. All-inorganic perovskite solar cell based on small amount of iodide CsPbBr_2.9_I_0.1_ as the absorber and *spiro*-OMeTAD as the HTM prepared in ambient condition achieved well-performance of 3.98 % efficiency, as opposed to 2.97 % for pure CsPbBr_3_ perovskite. Likewise, the addition of optimized amount of iodide (0.1 molar ratio) has successfully mitigated the hysteretic performance of CsPbBr_2.9_I_0.1_ perovskite solar cell, as compared to CsPbBr_3_ device. We concluded that there are two main factors to explain the origin of the *V*_oc_ in lead halide perovskite solar cells, which are the large band gap of the absorber and the deep hole selective transport material which are important for *V*_oc_ increment. Regarding the perovskite layer, improvement in the *V*_oc_ was observed when the band gap of the CsPbBr_3-*x*_I_*x*_ perovskite increases, independent of the HTM used. In particular, the increment of *V*_oc_ was more pronouncing for P3HT/MoO_3_-based solar cell, as opposed to the *spiro*-OMeTAD based solar cell due to lower work function property of P3HT/MoO_3_. Additionally, the *V*_oc_ was also affected by the selected HTM. When the *spiro*-OMeTAD was substituted by P3HT/MoO_3_, an increment of *V*_oc_ from 1.16 V to 1.3 V was obtained. However, *J*_sc_ was reduced due to the charge extraction issues, which is observable from the impedance spectroscopy measurements. Stability operation shows that the CsPbBr_2.9_I_0.1_ and CsPbBr_2.8_I_0.2_-based solar cells possessed better stability than the pure bromide and CsPbBr_2.9_I_0.1_ solar cells when *spiro*-OMeTAD was used as the HTM. Whilst, the perovskite solar cells showed no significant decay for the P3HT/MoO_3_ contact.

## Experimental section

### Materials

The fluorine-doped tin oxide (FTO) glass substrate with a size of 2.5 × 2.5 cm^2^ was purchased from Nippon Sheet Glass Co. Ltd (10 Ω/sq). The mesoporous titanium dioxide (mp-TiO_2_) was obtained from JGC Catalysts and Chemicals Ltd (PST-18NR) and was diluted with α-terpineol (Wako) (1:3 by weight). The perovskite films were prepared through two sequential deposition process. The pure CsPbBr_3_ perovskite film was prepared from a thin film of lead (II) bromide (PbBr_2_, > 98%) from Sigma-Aldrich. Additionally, small amount of lead (II) iodide (PbI_2_, > 98%) purchased from TCI Tokyo Chemical Industry CO., LTD, was added into the PbBr_2_ solution in order to synthesize a mixture of PbBr_2_:PbI_2_ in the molar ratio of 1:0, 1:0.1, 1:0.2, and 1:0.3. These solutions were dissolved with dimethlyformamide DMF (Sigma-Aldrich). Then, these films were dipped into a solution of cesium bromide (CsBr, 99.9%) obtained from Alfa Aesar, which dissolved in methanol (Wako) in a concentration of 15 mg/mL. For the hole transport materials (HTMs), 2,2′,7,7′-tetrakis[N,N-di(4-methoxyphenyl)amino]-9,9′-spirobifluorene, *spiro*-OMeTAD, obtained from Sigma-Aldrich was dissolved in chlorobenzene (purchased from Sigma-Aldrich). In order to improve its electrical properties, some additives were added, such as *bis* (trifluoromethane) sulfonimide lithium salt (Li-TFSI) dissolved with acetonitrile (520 mg/mL, Wako) and 4-*tert*-butylpyridine (TBP) which purchased from Sigma-Aldrich and TCI, respectively. Poly(3-hexylthiophene-2,5-diyl), P3HT, acquired from Sigma-Aldrich was dissolved in *o*-dichlorobenzene (Sigma-Aldrich) with a concentration of 17 mg/mL.

### Device fabrication

The FTO-coated glass was patterned using HCl (6 M)-etched with Zn powder and cleaned by firstly, ultrasonification in a basic bath of neutral water, acetone, isopropanol, and deionized water and secondly, plasma system for several minutes. A 20 nm of compact titanium dioxide (c-TiO_2_) prepared from titanium diisopropoxide bis (acetylacetonate) was deposited through atomic layer deposition method at 300 °C. The mesoporous layer mp-TiO_2_ was spin coated onto c-TiO_2_ surface at the spinning conditions of 500 rpm for 3 s and 6000 rpm for 30 s, followed by an immediate heating at 80 °C for 10 min, subsequently was calcined at 470 °C for 30 min. The synthesis of perovskite film was conducted via two-steps sequential method. Firstly, an aliquot of 90 μL PbBr_2_:PbI_2_, being a molar ratio of 1:0, 1:0.1, 1:0.2, and 1:0.3, was initially spin coated onto mp-TiO_2_ surface at 2500 rpm for 1 min. These films were heated at 75 °C for 30 min on a hot plate. Secondly, the PbBr_2_:PbI_2_ coated films were dipped into CsBr solution for 10 min at 50 °C, subsequently washed with 2-propanol solution for 1 min at 50 °C. The CsPbBr_3-*x*_I_*x*_ films were synthesized after annealing at 350 °C for 10 min. Subsequently, *spiro*-OMeTAD was spin coated on the surface of perovskite film at 500 rpm for 3 s, later 4000 rpm for 30 s. The films were left to dry inside a petri dish about 15 h in dark. No annealing treatments were required. On the other hand, the P3HT samples were prepared through spin coating process at 1000 rpm for 10 min. Drying procedure were finished after 16 h storage inside a petri dish in dark and then, an annealing treatment at 130 °C for 10 min was carried out. Finally, an 8 nm MoO_3_ film was thermally evaporated for P3HT-based solar cells, and 60 nm of Au was deposited for all devices.

### Materials characterization

The glass/FTO/CsPbBr_3-*x*_I_*x*_ substrates (where *x* varies between 0, 0.1, 0.2, and 0.3 in molar ratio) were characterized by several techniques. The UV-Visible spectra was performed via JASCO V-670 spectrophotometer from 200–900 nm. The emission spectra were obtained from JASCO FP- 6600 spectrofluorometer from 450–600 nm. The valence band of perovskite was measured through Ultraviolet Photo Electron Spectroscopy (UPS) (BUNKOUKEIKI). The X-Ray diffraction (XRD) was conducted through RINT-Ultima III, Rigaku in 2θ range from 14 to 50 degrees using monochromatic CuK_α_ radiation. The surface morphologies of the perovskite materials were investigated using a Quanta 400F FE-SEM equipped with an EDX feature.

### Device characterization

The current density-voltage (J-V) plot was measured by a solar simulator (CEP- 2000SRR, Bunkoukeiki Inc) with light source intensity of AM1.5G 100 mWcm^−2^, which was calibrated with a reference silicon cell. All solar cells were measured using a mask with active area of 0.12 cm^2^ under 1 sun. The photovoltaic measurements were carried out at a scan rate of 0.1 Vs^−1^ with 100 ms delay time and 10 mV voltage step. The measurement of external quantum efficiency (EQE) was performed through CEP-2000SRR, Bunko Keiki with 300 W Xe lamp. The monochromator was adjusted to 1 × 10^16^ mWcm^−2^ and was monitored by Si photodiode. The impedance spectroscopy at open circuit conditions was measured with a potentiostat (AUTOLAB PGSTAT204 connected to an impedance module FRA32M) in a range of frequencies from 1 MHz to 10 Hz in a perturbation amplitude of 0.01 V. Different light intensities were analyzed from 0.02 to 100 mWcm^−2^.

## Electronic supplementary material

Supplementary Information
