# Supplementary material for: Tunable Open Circuit Voltage by Engineering Inorganic Cesium Lead Bromide/Iodide Perovskite Solar Cells
Source: Sci Rep. 2018 Feb 6;8:2482. doi: 10.1038/s41598-018-20228-0 (PMC5802841; doi:10.1038/s41598-018-20228-0)
Supplement: Supplementary file 1 — Supplementary Information [file 41598_2018_20228_MOESM1_ESM.docx]

***Supporting Information***

**Tunable Open Circuit Voltage by Engineering Inorganic Cesium Lead Bromide/Iodide**

**Perovskite Solar Cells**

Chi Huey Ng,^1,2^ Teresa S. Ripolles,^2*^ Kengo Hamada,^2^ Siow Hwa Teo,^1,2^ Hong Ngee Lim,^1,3^ Juan Bisquert,^4,5*^ Shuzi Hayase^2*^

^1^Department of Chemistry, Faculty of Science, Universiti Putra Malaysia, 43400 UPM, Serdang, Selangor, Malaysia.

^2^Graduate School of Life Science and Systems Engineering, Kyushu Institute of Technology, 2-4 Hibikino, Wakamatsu-ku, Kitakyushu 808-0196, Japan.

^3^Functional Device Laboratory, Institute of Advanced Technology, Universiti Putra Malaysia, 43400 UPM, Serdang, Selangor, Malaysia.

^4^Institute of Advanced Materials (INAM), Universitat Jaume I, 12006 Castelló, Spain.

^5^Department of Chemistry, Faculty of Science, King Abdulaziz University, Jeddah, Saudi Arabia.





Figure S1. Ultraviolet photoelectron spectroscopy of CsPbBr_3-_*_x_*I*_x_* perovskite films on glass/FTO substrate.

**

**

Figure S2. XRD patterns of glass/CsPbBr_3-_*_x_*I*_x_*, where the amount of iodide varies as x = 0, 0.1, 0.2, and 0.3 in molar ratio. The theoretical peaks of pure perovskites of CsPbBr_3_ and CsPbI_3_ are also included.

**
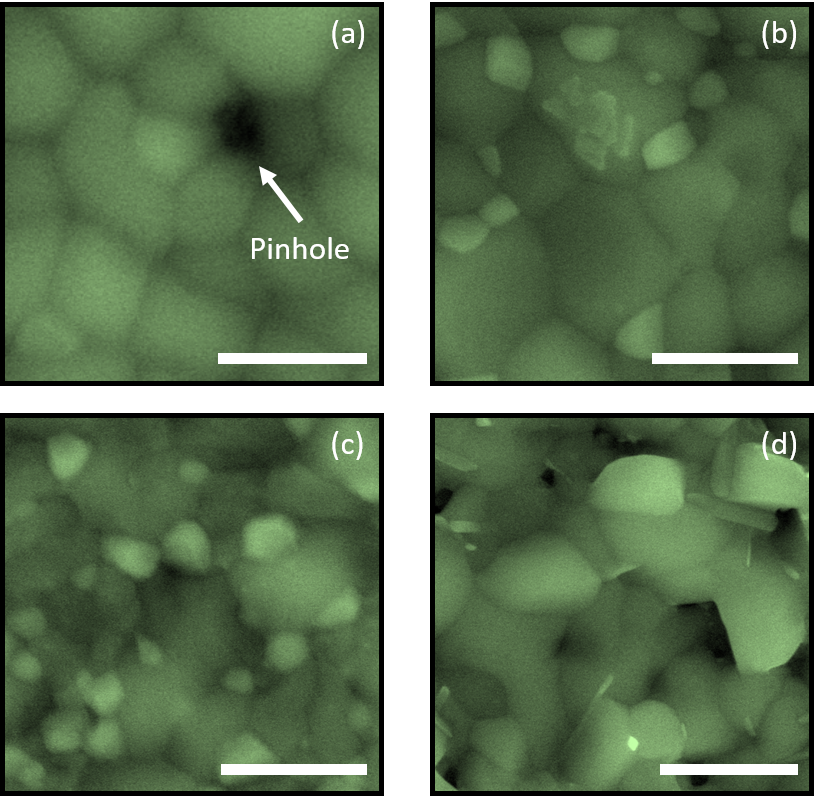
**

Figure S3. Top view FE-SEM images of (a) CsPbBr_3_, (b) CsPbBr_2.9_I_0.1_, (c) CsPbBr_2.8_I_0.2_, and (d) CsPbBr_2.7_I_0.3_ perovskite film. Bar size corresponds to 1 μm.

**20 µm**





Figure S4. Enlarged EQE spectrum of CsPbBr_3-_*_x_*I*_x_* perovskite solar cells shows the red-shifted EQE onsets.

**

**

Figure S5. J-V hysteresis performances of (a) CsPbBr_3_, (b) CsPbBr_2.9_I_0.1_, (c) CsPbBr_2.8_I_0.2_, and (d) CsPbBr_2.7_I_0.3_ perovskite solar cell with s*piro*-OMeTAD as the HTM. The dash lines show the reverse scan direction, while the solid lines represent the forward scan direction.





Figure S6. (a) J-V measurements under simulated AM 1.5G sun light of 100 mW cm^-2^ irradiance, and (b) under dark. (c) represents the EQE for glass/FTO/c-TiO_2_/mp-TiO_2_/CsPbBr_3-_*_x_*I*_x_*/P3HT/MoO_3_/Au solar cells, where *x* varies in 0, 0.1, 0.2, and 0.3 molar ratio.





Figure S7. Average photovoltaic performances where (a) *V*_oc_, (b) *J*_sc_, (c) *FF*, and (d) PCE and error bars of CsPbBr_3-_*_x_*I*_x_* perovskite solar cells with either *spiro*-OMeTAD (black column) or P3HT/MoO_3_ (red column) as HTMs.

**Table S1. Photovoltaic parameters of the CsPbBr_3-_*_x_*I*_x_* solar cells (*x* varies between 0, 0.1, 0.2, and 0.3 in molar ratio) with P3HT/MoO_3_ as the HTM. The photocurrent calculated from the integration of the EQE curves was also added.**

| Perovskite | HTM | V_oc_, V | J_sc_, mA cm^-2^ | FF | PCE, % | J_sc_[Cal.], mA cm^-2^ |
| --- | --- | --- | --- | --- | --- | --- |
| CsPbBr_3_ | P3HT/MoO_3_ | 1.30 | 3.80 | 0.62 | 3.07 | 5.35 |
| CsPbBr_2.9_I_0.1_ | P3HT/MoO_3_ | 1.23 | 3.80 | 0.54 | 2.51 | 5.41 |
| CsPbBr_2.8_I_0.2_ | P3HT/MoO_3_ | 1.17 | 3.39 | 0.51 | 2.01 | 5.40 |
| CsPbBr_2.7_I_0.3_ | P3HT/MoO_3_ | 0.99 | 3.40 | 0.53 | 1.76 | 4.87 |

**Table S2. Photovoltaic performances of champion CsPbBr_3-_*_x_*I*_x_* solar cells and average photovoltaic performances of five solar devices.**

| Solar cell | HTM | *V*_oc_, V | | *J*_sc_, mA cm^-2^ | | *FF* | | PCE, % | |
| --- | --- | --- | --- | --- | --- | --- | --- | --- | --- |
|  |  | Champion | Average | Champion | Average | Champion | Average | Champion | Average |
| CsPbBr_3_ | *spiro*-OMeTAD | 1.15 | 1.10±0.05 | 5.25 | 4.90±0.63 | 0.49 | 0.48±0.04 | 2.97 | 2.64±0.47 |
| CsPbBr_2.9_I_0.1_ | *spiro*-OMeTAD | 1.13 | 1.09±0.07 | 5.54 | 5.53±0.18 | 0.64 | 0.59±0.07 | 3.98 | 3.61±0.56 |
| CsPbBr_2.8_I_0.2_ | *spiro*-OMeTAD | 1.06 | 1.04±0.05 | 5.58 | 5.49±0.15 | 0.65 | 0.55±0.08 | 3.83 | 3.14±0.47 |
| CsPbBr_2.7_I_0.3_ | *spiro*-OMeTAD | 1.12 | 1.00±0.12 | 5.17 | 4.78±0.35 | 0.47 | 0.41±0.06 | 2.73 | 2.02±0.67 |
| CsPbBr_3_ | P3HT/MoO_3_ | 1.29 | 1.28±0.01 | 3.80 | 3.17±0.58 | 0.62 | 0.57±0.06 | 3.07 | 2.33±0.66 |
| CsPbBr_2.9_I_0.1_ | P3HT/MoO_3_ | 1.23 | 1.22±0.01 | 3.79 | 3.62±0.25 | 0.54 | 0.51±0.03 | 2.51 | 2.28±0.22 |
| CsPbBr_2.8_I_0.2_ | P3HT/MoO_3_ | 1.17 | 1.13±0.09 | 3.39 | 3.24±0.15 | 0.50 | 0.50±0.00 | 2.01 | 1.87±0.22 |
| CsPbBr_2.7_I_0.3_ | P3HT/MoO_3_ | 0.99 | 0.85±0.14 | 3.39 | 2.90±0.67 | 0.53 | 0.50±0.04 | 1.76 | 1.28±0.50 |
